# Effects of sitagliptin on intrahepatic lipid content in patients with non-alcoholic fatty liver disease

**DOI:** 10.3389/fendo.2022.866189

**Published:** 2022-08-22

**Authors:** Xingchun Wang, Bangfeng Zhao, Hang Sun, Hui You, Shen Qu

**Affiliations:** ^1^ Department of Endocrinology and Metabolism, Shanghai Tenth People’s Hospital, Tongji University, Shanghai, China; ^2^ Shanghai Center of Thyroid Diseases, Shanghai Tenth People's Hospital, School of Medicine, Tongji University, Shanghai, China

**Keywords:** intrahepatic lipid, nonalcoholic fatty liver disease, sitagliptin, metabolism, glucose

## Abstract

**Purpose:**

Dipeptidyl peptidase-4 inhibitors (DPP-4I), key regulators of the actions of incretin hormones, exert anti-hyperglycemic effects in type 2 diabetes mellitus (T2DM) patients. A major unanswered question concerns the potential ability of DPP-4I to improve intrahepatic lipid (IHL) content in nonalcoholic fatty liver disease (NAFLD) patients. The aim of this study was to evaluate the effects of sitagliptin on IHL in NAFLD patients.

**Methods:**

A prospective, 24-week, single-center, open-label, comparative study enrolled 68 Chinese NAFLD patients with T2DM. Subjects were randomly divided into 4 groups: control group who did not take medicine (14 patients); sitagliptin group who received sitagliptin treatment (100mg per day) (17 patients); metformin group who received metformin (500mg three times per day) (17 patients); and sitagliptin plus metformin group who received sitagliptin (100mg per day) and metformin (500 mg three times per day) (20 patients). IHL, physical examination (waist circumstances, WC; body mass index, BMI), glucose-lipid metabolism (fasting plasma glucose, FPG; hemoglobin A1c, Hb1A1c; triglycerides; cholesterol; alanine aminotransferase, ALT; aspartate aminotransferase, AST) were measured at baseline and at 24 weeks.

**Results:**

1) WC and BMI were decreased significantly in all groups except control group (all *P<*0.05). 2) There was no statistically significant difference in IHL among the sitagliptin, metformin, and sitagliptin plus metformin groups before and after treatment(all *P*>0.05). Only the metformin group showed a statistically significant difference in IHL before and after treatment(*P*<0.05). 3) Sitagliptin treatment led to a significant decrease in FBG and HbA1c when compared with the control group (all *P<*0.01). Additionally, HhA1c was significant decreased in the sitagliptin group when compared with the metformin group (*P*< 0.05). 4) HbA1c and FBG were decreased by 0.8% and 0.7 mmol/l respectively and the percentage of patients with HbA1c less than 7% was 65% with sitagliptin treatment.

**Conclusion:**

Sitagliptin improves abnormalities in glucose metabolism, but not reduces the IHL in T2DM with NAFLD, indicating that sitagliptin might be a therapeutic option for treatment of NAFLD indirectly while not directly on IHL. Clinical Trial Registration: https://clinicaltrials.gov/, identifier CTR# NCT05480007.

## Introduction

Non-alcoholic fatty liver disease (NAFLD) is highly associated with several components of metabolic syndrome (MS), particularly obesity, increased plasma lipid levels, insulin resistance, and type 2 diabetes mellitus (T2DM) (1–3). The proportion of NAFLD patients with concomitant obesity and T2DM is in the range of 25%-75% (4, 5). Diabetic patients with NAFLD may be existing insulin resistance and impaired lipid metabolism, and the insulin resistance may further increase fat deposition in the liver (6, 7), thereby exacerbating both conditions.

Lifestyle adjustments leading to weight loss are effective in the treatment of NAFLD and can also improve insulin sensitivity (8–10). The present therapeutic options used for treatment of NAFLD patients with diabetes include insulin sensitizing agents such as metformin and thiazolidinediones (11, 12). Metformin maintains glucose homeostasis by increasing the utilization of glycogen and inhibiting hepatic glucose output, and reduces the accumulation of fat mass in liver (11, 13). However, the effects metformin on liver histology is not clear and metformin is currently not recommended in treatment of NASH (non-alcoholic steatohepatitis). By activation of the peroxisome proliferator activated receptor (PPAR), thiazolidinediones increase insulin sensitivity, regulate lipid metabolism, alleviate liver damage, and decrease fat accumulation in liver (14, 15). However, they have not been able to improve the histological appearance of liver damage. No effective drug therapy for NAFLD has been established. Thus, given the difficulty in sustaining lifestyle modification, effective pharmacological options are necessary and incretin based therapies may represent an effective option.

Glucose dependent insulinotropic polypeptide (GIP) and glucagon like peptide 1 (GLP-1) are known incretine hormones. However, the metabolic effects of GIP are blunted in T2DM and only GLP-1 remains of interest in the treatment of T2DM and related disorders (16). Circulating GLP-1 is readily degraded by the enzyme dipeptidyl peptidase-4(DPP-4) (17–19). Therefore, DPP-4 inhibitors (DPP-4I) have been developed which can inhibit the degradation of GLP-1, thereby increasing incretin levels, stimulating insulin secretion, increasing sensitivity of beta cells to incretins, and increasing beta cell proliferation (20). DPP-4I can reduce fasting and postprandial plasma glucose levels (21, 22) and reduce HbA1c, although to a greater extent in Asian patients compared with Caucasians (23). Thus, these agents may be particularly useful for Chinese patients. Sitagliptin is a DPP-4 inhibitor which can increase insulin release and decrease glucagon levels by increasing the levels of active incretin (24–26).

The GLP-1 receptor (GLP-1R) has been found on human hepatocytes (27). Sitagliptin treatment has been shown to be safe, well tolerated, and have anti-diabetic effects. A previous study has shown that the change in HbA1c after stigliptin treatment for 8 and 12 weeks were significantly greater in T2DM patients with NAFLD than in T2DM patients without NAFLD (28). Forty-one patients with biopsy-proven NAFLD with T2DM were treated with sitagliptin (50 mg/day) for 12 months and showed a 0.7% reduction in HbA1c (29). Another study showed that HbA1c and fasting plasma glucose (FPG) in 20 NAFLD patients with T2DM who were treated with sitagliptin group were significantly decreased compared with a control group treated with diet and exercise (30). Four months of treatment with sitagliptin in NAFLD patients with T2DM diagnosed by ultrasonography led to significant decreased plasma glucose, HbA1c, aspertate aminotransferase (AST), and alanine transaminase (ALT) (31). However, body weight did not change in NAFLD patients with T2DM treated with sitagliptin (32). Animal experiments have shown a decrease in intrahepatic lipid content (IHL) after treatment with DPP-4I (33, 34). However, the effect of DPP-4I on hepatic fat accumulation has not been fully evaluated in NAFLD patients with T2DM. Therefore, the aim of this study was to investigate the effect of sitgliptin on IHL and glucose-lipid metabolism in Chinese NAFLD patients with T2DM.

## Methods

### Subjects

A total of 68 Chinese subjects were recruited from the outpatient department of Shanghai Tenth People’s Hospital. Informed consent was obtained from each participant after being informed of the aims of the study and potential adverse effects of the study drugs. This study was approved by the Ethics Committee of Shanghai Tenth People’s Hospital. Inclusion criteria were as follows: 1) age 30-70 years old, 2) fulfillment of the diagnostic criteria for T2DM by WHO in 1999 (HbA1c ranged from 7% to 10%, FPG< 11 mol/l, 2-hour blood glucose postprandial< 20mmol/l), 3) Fulfillment of the diagnostic criteria for NAFLD according to the guidelines of the Chinese Medical Association in 2010. The IHL content was measured by using 1H-magnetic resonance spectroscopy (1H-MRS) quantitative detection, and the liver fat content of the enrolled subjects was more than 5.5%. The subjects included in this study had either no history of alcohol consumption or their alcohol intake was less than 70 g/week in males and less than 140 g/week in females. Liver transaminase and serum creatinine were less than two times the upper limit of normal. The exclusion criteria were as following: 1) T2DM complicated with ketoacidosis, hyperosmolarity, acute and chronic infection, 2) serious heart, liver, kidney, lung disease, and liver damage, 3) alcoholic fatty liver, 4) drug use that influences glucose metabolism such as thiazide diuretics and hormones within three months, 5) Hypertension ≥ 180/110 mmHg, 6) gastrointestinal disease or absorption dysfunction, 7) recent trauma, surgery, or other conditions resulting in an increased stress response within the past three months. The clinical trial is NCT02118376.

### Anthropometric data measurement

Weight (Wt), height, waist circumference (WC), and calculated to body mass index (BMI, according to the formula:BMI (kg/m^2^) = body weight (kg)/height (m) ^2^) were measured for each subject at baseline as well as at 24 weeks after starting the trial.

### Lab testing

Venous blood was collected and serum was prepared after 8 hours of fasting before and after treatment. Total cholesterol (TCH), triglyceride (TG), low density lipoprotein cholesterol (LDL-C), high density lipoprotein cholesterol (HDL-C), ALT, AST, and alkaline phosphatase (AKP) were measured using an automatic biochemistry analyzer (model) at baseline and 24 weeks. FPG was measured using the glucose oxidase method. HbA1c was measured using the BIO-RAD VARIANTII.

### Measurement of liver fat content

To evaluate the liver fat content, HD magnetic resonance imaging (1.5 Tesla, GE) was performed at baseline and 24 weeks. The peak value of water proton peak, the area under the water proton peak (AUC-water), the peak value of fat peak and the area under the fat peak (AUC-IHL) were determined. The relative content of IHL was calculated by the following formula: IHL= AUC-IHL/(AUC-water+ AUC-IHL) ×100%.

### Grouping

Subjects were randomly divided into 4 groups using blocked randomization according to the drug treatment:

Group A: control group (14 patients, average age:53.4 ± 5.2 years old). The subjects received nothing at all. They were under diet and exercise control.
Group B: sitagliptin group (17 patients, mean age:54.4 ± 9.4 years old). These subjects received sitagliptin 100 mg per day.
Group C: metformin group (17 patients, mean age: 55.6 ± 10.9 years old). These subjects took metformin 500 mg three times per day.
Group D: sitagliptin plus metformin group (20 patients, mean age 54.5 ± 5.6 years old). These subjects took sitagliptin 100 mg per day and metformin 500 mg three times per day.

The treatment period was 24 weeks. All subjects were given health education regarding eating a diabetic diet and performing routine exercise during the treatment period. Adverse reactions and side effects of drug were monitored throughout the study. The primary endpoint was liver fat content and secondary endpoints including indicators of lipid (TCH, TG and LDL-C) (HDL-C), liver enzymes (ALT, AST and AKP) and glucose(FPG and HbA1c).

### Statistical analysis

All data are presented as mean ± standard deviation( $\bar{X}$ ±s). SPSS17.0 software was used for statistical analysis. Comparison of mean values was performed using a paired or unpaired *t*-test. Variance analysis (ANOVA) was utilized when comparing the difference among groups. LSD (Least-Significant Difference) was what we used for *post-hoc* tests in ANOVA. The statistical difference was considered significant for *P<* 0.05.

## Results

### The general clinical data

Self-pair comparison showed that BMI was significantly decreased with sitagliptin treatment(*P*< 0.05). Additional comparisons between the sitagliptin group and control group are displayed in **Table 1**. WC and BMI showed a statistically significant decrease comparing baseline with 24 weeks after treatment with sitagliptin, metformin, and metformin and sitagliptin (all *P<* 0.05). There was no statistically significant difference in WC and BMI at 24 weeks among the three groups(all *P* > 0.05) as shown in **Table1**.

**Table 1:** Comparison of the clinical characteristics between groups.

Group		At baseline	At 24 weeks
A(control)	Sex	14(male/female:8/6)	14(male/female:8/6)
	Age (years old)	53.40 ± 5.20	53.40 ± 5.20
	BMI (kg/m^2^)	24.06 ± 2.85	23.85 ± 2.74
	Waistline(cm)	83.0 ± 6.5	81 ± 6.87
B(sitagliptin)	Sex	17(male/female:10/7)	17(male/female:10/7)
	Age (years old)	54.40 ± 9.0	54.40 ± 9.40
	BMI (kg/m2)	25.41 ± 3.45	24.39 ± 3.02※
	Waistline(cm)	86.9 ± 9.8	84.88 ± 8.19※
C(metformin)	Sex	17(male/female:9/8)	17(male/female:9/8)
	Age (years old)	55.63 ± 10.9	55.63 ± 10.9
	BMI (kg/m^2^)	26.46 ± 2.86	25.75 ± 3.55※
	Waistline(cm)	88.50 ± 8.00	88.67 ± 8.80※
D(Sitagliptin+ metformin)	Sex	20(male/female:9/11)	20(male/female:9/11)
	Age (years old)	54.55 ± 6.6	54.55 ± 6.6
	BMI (kg/m^2^)	26.07 ± 3.24	25.53 ± 3.02※
	Waistline(cm)	90.0 ± 8.20	86.8 ± 6※

Self-pair comparison before and after treatment, ※P<0.05.

Data are number of patients or mean ± standard deviation.

BMI, body mass index.

### Liver fat content

The average IHL content was 61.88%, the highest IHL was 86.67% and the lowest IHL was 22.5% in sitagliptin group at baseline. The average IHL content was 42.11% in the sitagliptin group at 24 weeks which showed a trend decrease compared with baseline without statistical significance (*P* > 0.05). The average IHL was 54.43% at baseline and was 47% at 24 weeks in the control group. There was no significant statistical significance between sitagliptin group and the control group as shown in **Table 2**. IHL content was significantly decreased in metformin group (from 68.73 ± 14.6 to 32.57 ± 16.7) (*P*< 0.05). Data for liver function tests are shown in **Table 2**. ALT and AST were not significantly different among the groups before treatment, however after treatment, ALT and AST in the sitagliptin group was significnalty higher compared with the metformin group (all *P<* 0.05).

**Table 2:** Comparison of the intrahepatic lipid and liver enzymes between groups.

Group		At baseline	At week 24
A(Control)	The maximum fat content(%)	84.74%	67.2%
	The minimum fat content(%)	6%	6.8%
	The average fat content(%)	54.43%	47.7%
	Fat content of male(%)	56.4%	49.6%
	Fat content of female(%)	52.4%	45.6%
B(Sitagliptin)	The maximum fat content(%)	86.67%	91.20%
	The minimum fat content(%)	22.5%	7.10%
	The average fat content(%)	61.88%	42.11%
	Fat content of male(%)	60%	40.18%
	Fat content of female(%)	64%	41.97%
	ALT(U/L)	26.81 ± 12.68	26.23 ± 17.33 △
	AST(U/L)	21.76 ± 5.02	22.00 ± 8.24 △
	AKP(U/L)	75.62 ± 22.49△	77.15 ± 37.94
C(metformin)	The average fat content(%)	68.73 ± 14.6	32.57 ± 16.7※
	ALT(U/L)	24.00 ± 9.53	22.00 ± 12.57※
	AST(U/L)	19.25 ± 3.62	21.20 ± 8.50
	AKP(U/L)	90.75 ± 14.87	71.20 ± 32.38
D(sitagliptin+ metformin)	The average fat content(%)	65.88 ± 16.4	42.7 ± 22.5
	ALT(U/L)	25.45 ± 14.45	25.14 ± 15.99
	AST(U/L)	22.00 ± 8.75	21.14 ± 7.27
	AKP(U/L)	64.18 ± 19.59△	59.00 ± 20.39

Self-pair comparison before and after treatment, ※P<0.05;vs Group C, △P<0.05.

ALT, alanine transaminase; AST, aspertate aminotransferase; AKP, alkaline phosphatase.

### Glucose-lipid metabolism

Self-pair comparison showed that FBG and HbA1c were significantly decreased after 24 weeks treatment with sitagliptin (all *P<* 0.05). Additionally, FBG and HbA1c were significantly decreased in the sitagliptin group when compared with the control group (all *P<* 0.01) as shown in **Table 3**. There was a significant decrease in HbA1c in tbe sitagliptin group compared with the metformin group (all *P*< 0.05). However, the sitagliptin group had a higher HbA1c and FBG compared with the sitagliptin plus metformin group after the 24 week treatment period (all *P*<0.05) as shown in **Table 3**. HbA1c was decreased by 0.8% and FBG was decreased by 0.7mmol/l with sitagliptin treatment while HbA1c was decreased by 0.3% and FBG was decreased by 0.7mmol/l with metformin treatment. Additionally, the percentage of HbA1c less than 7% was 65% and 25% after sitagliptin and metformin treatment, respectively. HbA1c was decreased by 1% and FBG was decreased by 1.3mmol/l, and the percentage of HbA1c level that less than 7% was 72.7% after sitagliptin plus metformin treatment. TCH, TG, HDL-C, LDL-C showed no significant difference among the groups before or after treatment. There was a trend toward a decrease in TG and HDL in the metformin group after treatment but this did not reach statistical significance (all *P*>0.05).

**Table 3:** Comparison of the glucose-lipid data between groups.

Group		At baseline	At week 24s
A(control)	FPG(mmol/l)	9.45 ± 1.83	9.96 ± 2.55
	HbA1c(%)	8.08 ± 1.13	8.16 ± 1.37
	TCH(mmol/l)	4.81 ± 0.95	4.56 ± 0.55
	HDL(mmol/l)	1.22 ± 0.17	1.20 ± 0.24
	TG(mmol/l)	1.73 ± 0.93	1.59 ± 1.04
	LDL(mmol/l)	2.92 ± 1.02	2.92 ± 1.02
B(sitagliptin)	FPG(mmol/l)	8.39 ± 1.89	7.60 ± 1.55※*
	HbA1c(%)	7.93 ± 0.91	6.95 ± 0.86※*△
	TC(mmol/l)	5.22 ± 0.92	5.19 ± 0.95
	HDL(mmol/l)	1.31 ± 0.38	1.31 ± 0.50
	TG(mmol/l)	1.70 ± 0.82	1.67 ± 1.08
	LDL(mmol/l)	3.02 ± 0.97	3.02 ± 0.97
C(metformin)	FPG(mmol/l)	10.40 ± 2.46	9.71 ± 2.16
	HbA1c(%)	8.60 ± 1.17	8.38 ± 0.61
	TCH(mmol/l)	4.88 ± 0.64	4.84 ± 1.06
	HDL(mmol/l)	1.18 ± 0.31	1.20 ± 0.28
	TG(mmol/l)	1.94 ± 0.97	1.69 ± 0.83
	LDL(mmol/l)	2.83 ± 0.65	2. 90 ± 0.26
D(sitagliptin+ metformin)	FPG(mmol/l)	9.60 ± 2.03	8.33 ± 1.63△
	HbA1c(%)	7.83 ± 0.58	7.04 ± 0.48△
	TCH(mmol/l)	4.46 ± 1.03	4.53 ± 1.02
	HDL(mmol/l)	1.34 ± 0.27	1.37 ± 0.27
	TG(mmol/l)	1.26 ± 0.62	1.35 ± 0.51
	LDL(mmol/l)	2.54 ± 1.01	2.54 ± 0.90

Self-pair comparison before and after treatment, ※P<0.05;vs Group A, *P<0.05; vs Group B, #P<0.05; vs Group C, △P<0.05: vs Group D, ※P<0.05.

FBG, fasting plasma glucose; HbA1c, hemoglobin A1c; TCH, total cholesterol; HDL, high density lipoprotein cholesterol; TG, triglyceride; LDL, low density lipoprotein cholesterol.

## Discussion

The prevalence of NAFLD is increasing with the rapid development of modern economies which permit lifestyles contributing to the development of numerous metabolic disorders and making NAFLD one of the most serious chronic diseases globally (35). NAFLD has been associated with vascular endothelial dysfunction, which is an indicator of the early stages of arteriosclerosis (36). The thickness of the carotid artery intima-media and the plaque formation rate in NAFLD patients were higher compared with controls (37). It was further shown that, the risk of cardiovascular disease in NAFLD patients is 5.3 times higher than in controls, and the incidence of diabetes, hypertension, systemic inflammatory response and metabolic syndrome are 15 times higher than that of the control group (38). Therefore, an effective treatment is needed to improve the underlying disease process of patients with NAFLD. A previous study has shown that the effects of dietary intervention using a carbohydrate (CH)-restricted low energy diet (CH: 10% of total energy) for 48 hours lowered IHL content in obese subjects compared with a high CH diet (CH: 65% of total energy) (39). The effect of diet on reducing IHL content can also be found in T2DM patients or obese subjects with impaired glucose tolerance (IGT) (40–42). In this study, we explored the effects of sitagliptin on IHC lipid content and metabolic profile in NAFLD patients with T2DM.

Liver fat content measured by magnetic resonance 1H-MRS has been shown to be reliable and has been accurately replicated. The diagnosis of fatty liver is based on the area under lipid peak/AUC-IHL + AUC-water≥ 5.5% or area under lipid peak/area under water peak≥20% (43, 44). The liver fat content was about 65% and ranged from 6% to 89.7% in all these subjects. Many of the subjects in this study had never been shown to have fatty liver by abdominal ultrasound prior to entry into the study. 1H-MRS can detect mild fatty liver with a much higher sensitively than conventional ultrasonography. Utilizing 1H-MRS in our study, the average fat content of all subjects was about 65%, which classifies most patients as having moderate or severe fatty liver.

The mechanism of occurrence and development of NAFLD is not completely understood. One commonly proposed theory suggesting a “two hit” mechanism: too much triglycerides stored in the liver cells constitutes the first hit, and this contributes to the second hit which may be a variety insults such as insulin resistance, oxidative stress, inflammatory reaction, lipid peroxidation, etc (45). Logistic regression analysis also found that insulin resistance (HOMA-IR), waist to hip ratio, total cholesterol, and triglycerides are independent risk factors for NAFLD (46). Insulin resistance is thought to be one of the key initial pathogenic mechanisms contributing to the development of NAFLD as high insulin levels can result in liver fat deposition by stimulating the oxidation of surrounding adipose tissue resulting in increased levels of serum free fatty acids (FFA), increased removal of serum FFA through increased hepatic storage, and increasing endogenous synthesis of FFA in the liver. Furthermore, deposition of FFA in the liver also exacerbates insulin resistance through increase fatty acid oxidation and free radical production leading to a vicious cycle of worsening liver fat accumulation and insulin resistance (47). Some adipocytokines such as adiponectin, leptin and resistin play important roles in the development of NAFLD by acting on liver by producing increased inflammation reaction and increasing insulin resistance (48, 49).

It is believed that both insulin resistance and hypertriglyceridemia play important roles in the development of diabetes and NAFLD. Currently, the treatment of patients with diabetes and NAFLD includes the use of insulin sensitizing agents such as metformin and thiazolidinediones (11, 12). Metformin improves glucose homeostasis by increasing the utilization of glycogen and inhibiting glucose output. Additionally, metformin reduces the accumulation of fat in the liver (11, 13). A previous study has shown that metformin (750 mg/day) reduced IHL content by 25% in obese patients with T2DM (50). However, metformin has no significant effect on liver histology and is not recommended for the treatment of NASH. A recent meta-analysis suggested that the use of thiazolidinediones may improve hepatic steatosis both in non-diabetic and diabetic patients (51). However, it exerts limit actions in histological liver injury. Thus, we studied the effects of DPP-4 inhibitors on intrahepatic lipid content and the metabolic profile in NAFLD patients with T2DM. There was a trend toward a reduction in liver fat content with sitagliptin treatment. The average liver fat content was decreased from 61.88% to 42.11% after sitagliptin treatment, although this did not reach statistical significance. The lack of statistical significance may be due to the small sample size and short treatment period among other factors. Additionally, the peak plasma level of active GLP-1 was 15-20 pmol/L after treatment with sitaglitin while the GLP-1 receptor agonist lirglutide led to higher levels (4000-8000 pmmol/L) (52, 53). Thus, the lack of statistical significance observed in the reduction of IHL content may be due to the relatively lower plasma concentrations of GLP-1 achieved with sitagliptin. Metformin treatment resulted in a significant reduction in liver fat content, which is likely due to activation of hepatic AMP-activated protein kinase (AMPK) by metformin which suppresses hepatic TG production and increases hepatic catabolic metabolism (54). Previous studies have also suggested that AMPK may be able to be directly activated by GLP-1 (55).

The GLP-1 receptor belongs to the G protein coupled glucagon receptor family and is widely distributed throughout the body and can be found in the pancreas, gastrointestinal tract and other tissues (56). Studies in animal models have shown that DPP-4 inhibitors can inhibit DPP-4 activity by 90% and increase plasma concentrations of exogenous GLP-1 thereby decreasing postprandial blood glucose (56, 57). Previous studies have shown that HbA1c was decreased by 0.65 percentage points with sitagliptin treatment after 24 weeks poorly controlled T2DM patients (24–26). In this study, HbA1c was decreased by 0.8 percentage points, and FBG was decreased by 0.7 mmol/l in the sitagliptin group while HbA1c was decreased by 1 percentage point and FBG was decreased by 1.3 mmol/l in the sitagliptin plus metformin group. HbA1c significantly decreased in the sitagliptin group was significant compared with the metformin group. The percentage of HbA1c level less than 7% was 65%, 25%, and 72.7% after sitagliptin, metformin and sitagliptin plus metformin treatments, respectively. These results suggest that sitagliptin effectively decreases blood glucose levels. There were no adverse reactions of hypoglycemia during the treatment period. Each participant demonstrated good compliance, and the treatment was well tolerated. Animal studies have confirmed that DPP-4 inhibitors can reduce blood glucose and triglyceride levels significantly in high fat diet (HFD) fed diabetic rats (58). However, in our study, the effects of sitaglipin and metformin on lipid metabolism were not obvious. This may be due to relatively short duration of treatment used in this study.

The present study also had some limitations. Firstly, the number of patients included in this study was relatively small and the study period also was relatively brief. Secondly, the change of total fat mass and distribution of fat was not measured in this study. Therefore, we cannot estimate the effects of sitagliptin on fat redistribution. A large-scale, longer-term study clinical trial is warranted to verify our findings.

In conclusion, our study showed that 24 weeks of treatment with sitagliptin was safe and well tolerated. It improves glycemic control and can slightly reduce liver fat content in NAFLD patients with T2DM. Therefore, sitagliptin may have therapeutic potential for NAFLD patients was well as those with other metabolic disorders indirectly while not directly on IHL.

## Data availability statement

The raw data supporting the conclusions of this article will be made available by the authors, without undue reservation, to any qualified researcher.

## Ethics statement

The studies involving human participants were reviewed and approved by the ethics committee of Shanghai Tenth People’ s Hospital. The patients/participants provided their written informed consent to participate in this study.

## Author contributions

XCW and BFZ performed the experiment and draft the manuscript. HS involved in review and revised the manuscript. HY participated in the data collection. SQ designed the study in the revised manuscript.

## Funding

The research was supported by the Climbing Talent Program of Shanghai Tenth People’s Hospital(2021SYPDRC047) and National Nature Science Foundation of China(NO.81900781).

## Conflict of interest

The authors declare that the research was conducted in the absence of any commercial or financial relationships that could be construed as a potential conflict of interest.

## Publisher’s note

All claims expressed in this article are solely those of the authors and do not necessarily represent those of their affiliated organizations, or those of the publisher, the editors and the reviewers. Any product that may be evaluated in this article, or claim that may be made by its manufacturer, is not guaranteed or endorsed by the publisher.
